# The effect of university students’ violence tendency on their attitude towards domestic violence and the factors affecting domestic violence attitudes

**DOI:** 10.5249/jivr.v12i1.1224

**Published:** 2020-01

**Authors:** Ruken Yagiz, Ümran Sevil, Özlem Guner

**Affiliations:** ^*a*^ Faculty of Nursing, Ege University, İzmir,Turkey.; ^*b*^ School of Health, Sinop University, Sinop,Turkey.

**Keywords:** Student, Violence tendency, Domestic violence

## Abstract

**Background:**

The aim of this study was to investigate the tendency of violence and the factors affecting their attitudes towards domestic violence in university students studying health sciences.

**Methods:**

The research was conducted with a total of 318 voluntary students studying in the senior year of nursing department in Faculty of Nursing, Ege University, Turkey and bearing the appropriate criteria for the participation and data were collected in the period June-July 2017. “Personal Information Form”, “Violence Tendency Scale (VTS)” and “Domestic Violence Attitude Scale (DVAS)” developed by the researchers have been used in data collection.

**Results:**

The research was conducted with a total of 318 students with average age of 22.41±1.49. Av-erage VTS scores of the students were found to be 28.13±6.28. The factor effective on violence tendency has been determined to be the student’s sex. DVAS of the students has been found to be 19.90±6.76.

**Conclusions:**

Violence phenomenon directed to youth was conducted on senior university students being a more developed group socially. However, young people’s exposure to violence was found high (42.8%). Nevertheless, their scores on violence tendencies and domestic violence attitude were ascertained low.

## Introduction

Violence is the intense and destructive occurrence of the feeling of hostility and anger towards people and objects.^1^ World Health Organization defines violence as “the intentional use of physical force or power, threatened or actual, against oneself, another person, or against a group or community, that either results in or has a high likelihood of resulting in injury, death, psychological harm, maldevelopment, or deprivation”.^2^ As a general perception, violence can be viewed as a sanction that people apply to each other. Along with the formation of coexistence culture, the perception of power pave the way for people to apply violence to one another.^3^ Violence is a multifaceted problem related to individual thoughts, attitudes and behaviors. Various factors including biological, psychological, social, cultural, economic and political ones may affect the formation of violence.^4,5^

Violence continues to exist as one of the social problems of people in the developed and developing countries of the 21st century.^2,6,7^ It has been determined that more than two million people in the world are hurt from the violence and suffering from both physical and emotional permanent disabilities every year.^2,8,9^ Although violence has been stated to be increasing in the studies conducted in Turkey,^8,9^ data to represent the whole country has not been reached. On the other hand, violence is analyzed within the context of domestic violence.^10-12^ However, violent incidents today are often experienced among adolescents, especially in schools and they increasingly reach to worrisome dimensions. For this reason, there is a need for the studies directed to solving the violence problem in the society particularly in the schools where our children receive education.^1^ It is well-known that violence is increasing both in the world and our country and this increase becomes disconcerting.^2,7,8^ It is also highlighted in many resources that special effort should be put forth in this matter. It is assumed that university students are an important group in the sense of increasing their awareness for violence and its prevention methods as they are the future parents who will generate a healthy family and healthy society. The role of nurses in determination and prevention of violence cannot be denied. In this regard, it is inevitable for nurses being in charge of the protection and improvement of community health to take responsibility in prevention of violence. Nurses have a special position in determining the children and families bearing the risk of violence, providing the necessary assistance to the child applying or being exposed to violence and finding acceptable solutions to the situation.^13^

## Materials and Methods

**1. Setting**

The research was conducted with a total of 318 voluntary students studying in the senior year of nursing department in Faculty of Nursing, Ege University, Turkey and bearing the appropriate criteria for the participation and data were collected in the period June-July 2017. 

**2. Sample Selection **

Research population is composed of senior students (393 students) studying in Faculty of Nursing, Ege University. Every student to be included in the research participated in the sample once. Sample selection wasn’t made and it was planned to apply to the whole population. However, the research was completed with 318 students meeting the sampling criteria which corresponded to 80.91% of the population. 

**Sampling criteria;**

• Studying in Faculty of Nursing, Ege University

• Being a nursing student,

• Being a senior student in the relevant institution after starting collection of research data, 

• Speaking and understanding Turkish,

• Being open to communication and psychologically and mentally healthy,

• Volunteering for the research.

**3. Research Questions **

Do violent tendencies of university students effect domestic violence attitudes?

Do socio-demographic characteristics of university students affect domestic violence attitudes?

**4. Data Collection **

Data were collected during June-July 2017 through face-to-face interviews conducted by researchers for five weekdays. 

**5. Data Collection Tools **

Personal Information Form, Violence Tendency Scale and Domestic Violence Attitude Scale developed by the researchers were used in data collection.

**Personal Information Form **

Personal Information Form was prepared by the researcher in line with the literature.^14,15^ The personal information form consists of eight questions. While the information of participants on their age, sex, parents’ educational background, family income, being exposed to or witnessing domestic violence were queried in the questionnaire, there were also some other questions like the type of violence exposed or witnessed. 

**Violence Tendency Scale**

Violence Tendency Scale (VTS) was developed by Haksan and Yıldırım in 2012. There are 20 items in this scale. The alternatives for each item are “never”, “sometimes” and “always”. The scale gets a maximum of 60 points. Cronbach-alpha reliability coefficient of the scale was established as 0.87.^16^ The value of 0.74 was determined to be the Cronbach-alpha reliability coefficient of the scale. 

**Domestic Violence Attitude Scale**

Domestic Violence Attitude Scale was developed by Şahin and Dişsiz in 2009. The scale is composed of 13 items and there are 5 alternatives for each item. These are as follows; “Strongly disagree (1), Disagree (2), Indecisive (3), Agree (4), Strongly Agree (5)”. The scale gets a maximum of 65 points. The scale is composed of both named and summable 4 factors. The scale consist of four sub-dimension. These sub-dimensions “the normalization of violence sub-dimension (1,2,3,4,5 dimension)”, “the generalization of violence sub-dimension (6,7,8 dimension)”, “the causation of violence sub-dimension (9,10,11 dimension)” and “ the concealment of violence sub-dimension (12,13 dimension)”. Considering the additivity and direction, it can be used unidimensionally. 0.72 was determined as the Cronbach-alpha reliability coefficient of the scale.^17^ The value of 0.75 was determined to be Cronbach-alpha reliability coefficient of the scale.

**6. Ethical Considerations **

In order to perform the research, ethics committee approval No:92112210-050.05.04 dated:28.03.2017 was received from Ege University Committee on Scientific Research and Publication Ethics. For performing the research, required institutional permission was obtained from Faculty of Nursing, Ege University and permission for use of scale from the researchers who developed the scales. In addition, students were informed about the purpose of the study, the benefits to be obtained from the research, the time they would spend on the interview and their verbal approvals were taken. 

**7. Data Analysis **

Data obtained from the research were evaluated with Statistical Package for the Social Sciences (SPSS) 20. Data assessment and analysis have been performed by using Kolmogorov normal distribution test, number-percentage distribution, Spearman correlation, Mann Whitney-U and Kruskal Wallis tests. The results were accepted within the confidence interval of 95% at p<0.05 significance level. 

## Results

The research was conducted with 318 students (male:44, female: 274) with the means age of 22.41±1.49. 80.91% of the population (318 students) was reached. Sosciodemographic factors were showen in Table 1.

**Table 1:** Mean Scores, Standard Deviations and Test Values belonging to the violence tendencies and domestic violence attitudes of university stu-dents by their socio-demographic characteristics.

		VTS				DVA			
**Sex**	**n**	**X**	**SD**	**U**	**p**	**X**	**SD**	**U**	**p**
Female	274	27.29	5.72	2866.000	0.000	19.03	5.6	3516.000	0.000
Male	44	33.36	7.07			25.34	10.14		
**Mother’s education**	**n**	**X**	**SD**	**Chi-square**	**p**	**X**	**SD**	**Chi-square**	**p**
Illiterate	19	28.57	5.94	1.185	0.946	21.84	8.3	3.675	0.597
Literate	13	28.69	8.34			20.76	7.01		
Primary school	169	28.13	6.16			19.93	7.29		
Secondary school	32	27.46	5.66			18.56	4.81		
High school	63	28.33	6.29			20.34	6.21		
University	22	27.81	7.46			18.18	4.39		
**Father’s education**	**n**	**X**	**SD**	**K-W**	**p**	**X**	**SD**	**K-W**	**p**
Illiterate	4	34.75	11.84	5.744	0.332	27.25	10.4	9.533	0.090
Literate	6	25.66	8.4			17.83	4.26		
Primary school	113	27.69	5.37			19.60	8.01		
Secondary school	61	28.18	7.11			20.27	5.58		
High school	87	28.13	6.07			20.33	5.86		
University	47	28.89	6.63			19.00	6.17		
**Family Income**	**n**	**X**	**SD**	**K-W**	**p**	**X**	**SD**	**K-W**	**p**
Good	43	26.41	4.12	2.589	0.274	19.53	6.2	0.084	0.959
Moderate	264	28.40	6.45			19.96	6.9		
Bad	11	28.36	8.13			19.90	5.87		
**Being Exposed to Violence **	**n**	**X**	**SD**	**K-W**	**p**	**X**	**SD**	**K-W**	**p**
Yes	111	30.56	6.47	8.658	0.013	19.43	6.04	2.668	0.263
No	182	27.45	6.11			20.07	7.38		
Rarely	25	28.71	6.00			20.76	4.88		
**Witnessing Violence **	**n**	**X**	**SD**	**K-W**	**p**	**X**	**SD**	**K-W**	**p**
Yes	136	28.26	6.24	16.773	0.001	19.48	5.94	11.809	0.003
No	144	27.09	5.75			19.68	7.64		
Rarely	38	31.63	7.12			22.26	5.55		
**Type of Violence **	**n**	**X**	**SD**	**K-W**	**p**	**X**	**SD**	**K-W**	**p**
Physical	66	28.83	5.76	1.113	0.774	19.59	6.08	1.635	0.652
Emotional	47	28.74	6.01			20.08	5.27		
Economic	13	29.30	7.11			19.84	7.79		
Verbal	61	29.36	6.76			20.54	6.14		

X: Mean, SD: Standart Deviation, K-W: Kruskal Wallis, U: Mann Whitney-U.

VTS score means of university students were found as 28.13±6.28. Means scores of Domestic Violence Attitude Scale were the 19.90±6.76.

Concerning the participants’ sub-dimension scores of the domestic violence attitudes, it was established that the mean score for the normalization of violence sub-dimension was 6.67+2.59, the mean score for the generalization of violence sub-dimension was 4.07+1.72, the mean score for the causation of violence sub-dimension was 5.88+2.15 and the mean score for concealment of violence sub-dimension was 3.27+1.81. The high means in the sub-dimensions of the scale indicated the attitude towards high domestic violence. Accordingly, the means for the normalization of violence was the highest means and followed by the causation of violence and generalization of violence, respectively. Concealment of violence was the lowest means. According to the analysis results, there was a significant difference between the violence tendencies by sex (u (318) = 2866.000, p= .000) and domestic violence attitude scores (u (318) = 3516.000, p= .000). It was ascertained that the violence tendency scores of the male students (Mean = 33.36+7.07) was higher than the score of female students (Mean= 27.29+5.72). When the domestic violence attitude scores were investigated, the scores of male students (Means = 25.34+10.14) was higher than the female students (Mean =19.03+ 5.6) (Table 1). Briefly, males had higher domestic violence attitudes and violence tendencies scores than females. Due to the number of male students in the study was lower than the female, so we could not generalize.

The differences between domestic violence attitude scores and violence tendencies of the university students by the mother’s educational background were determined by Kruskal Wallis h analysis. The mother’s educational did not lead to a significant difference in the violence tendency scores of university students by (Chi-square (n= 318) = 3.936, p= .559) and domestic violence attitude scores (Chi-square (n= 318) = 1.185, p= .597) (Table 1).

Kruskal Wallis h analysis was used to the purpose of determining whether there was a difference between domestic violence attitude scores and violence tendencies of the university students by the father’s educational. The father’s educational did not lead to a significant difference in the violence tendency scores of university students by (Chi-square (n= 318) = 5.744, p= .332) and domestic violence attitude scores (Chi-square (n= 318) = 9.533, p= .090) (Table 1).

Kruskal Wallis h analysis was used to the purpose of determining whether there was a difference between domestic violence attitude scores and violence tendencies of the university students with the monthly income of the family. The monthly income of the family did not lead to a significant difference in the violence tendency scores of university students by (Chi-square (n= 318) = 2.589, p=0.274) and domestic violence attitude scores (Chi-square (n= 318) = 0.084, p= .959). 

Kruskal Wallis h analysis was used to investigate if there was a difference between domestic violence attitude scores and violence tendencies of the university students by being subject to domestic violence. Although domestic violence attitude scores (Chi-square (n= 318) = 2.668, p= .263) was no different significantly by being subject to domestic violence, violence tendency scores were observed to differ significantly (Chi-square (n= 318)= 8.653, p= .013) (Table 1). The differences violence tendency scores during the subject to domestic violence were tested by Mann Whitney U analysis and the results showed that there was a significant difference was ascertained between “the individuals exposed to domestic violence”, “the individuals rarely exposed to domestic violence” and “the individuals with no exposure to domestic violence”. This differentiation was in favor of “the individuals exposed to domestic violence” and “the individuals rarely exposed to domestic violence”. In this sense, it can be said that the violence tendencies of the individuals who was exposed to violence was high. 

Kruskal Wallis h analysis was used to the purpose of determining whether there was a difference between domestic violence attitude scores and violence tendencies of the university students by witnessing domestic violence. Violence tendency scores by witnessing domestic violence (Chi-square (n= 318)= 16.773, p= .001) and the attitude scores of domestic violence (Chi-square (n= 318)= 11.809, p= .003) differ significantly (Table 1). As a result of the Mann Whitney U analysis performed to understand the reason for this difference, a significant difference was ascertained between “the individuals exposed to domestic violence”, “the individuals rarely exposed to domestic violence” and “the individuals with no exposure to domestic violence” in terms of the violence tendencies. This differentiation was found in favor of “the individuals witnessing domestic violence” and “the individuals rarely witnessing domestic violence”. In other words, the violence tendencies of the university students witnessing domestic violence and rarely witnessing domestic violence and their attitude towards domestic violence were found higher than those not witnessing domestic violence. 

The differences between domestic violence attitude scores and violence tendencies of the university students by the type of domestic violence exposed to or witnessed were determined by Kruskal Wallis h analysis. It was determined following the analysis that the violence tendencies of university students by the type of domestic violence exposed to or witnessed (Chi-square (n= 318)=1.113, p=0.774) and their domestic violence attitude scores (Chi-square (n= 318)= 1.635, p= .652) did not different statistically (Table 1).

As a result of the Spearman Rank correlation analysis performed with the purpose of determining whether there was a relation between the violence tendencies of university students and their domestic violence attitudes, it was determined that there was a linear and significant relation between the violence tendency scores of university students and their domestic violence attitude scores (r= +0.308, p= .000). Accordingly, the higher the violence tendency scores of university students were, the higher their domestic violence attitude scores become. 

## Discussion

In this study, the purpose of the interview form is to reveal the violence tendencies of nursing students studying in Faculty of Nursing, Ege University and to analyze the relation of these data with the demographic variables and domestic violence attitude.

According to the results of the research, it has been determined that there is a linear and significant relation between the violence tendency scores of university students and their domestic violence attitudes. In this regard, the higher the violence tendency scores of university students are, the higher their domestic violence attitude scores become. When violence tendency and domestic violence attitude are analyzed by sex, averages of males were higher than females. This finding complies with the results of other studies in the literature.^18-21^ These differences in the tendencies and attitudes for violence can be explained with gender socialization. Gender socialization is generally expressed as males’ learning masculine behaviors and females’ learning feminine behaviors.^22^ The origins of gender differences in human behavior are primarily based on evolved trends in differences between genders or in differentiated positions of women and men in social structures.^23^ Gender roles focus on social roles. Gender roles are defined as the expectations shared on the basis of the sex of individuals who define themselves only as social. ^24^ In this regard, men are expected to be more venturous and aggressive while women are expected to be more submissive and timid. 

There was no significant difference in terms of violence tendencies and domestic violence attitudes of university students by the educational background of parents. As a result of the study conducted by Birnbaum et al. (2017) in Canada with 353 children, it has been stated that domestic violence isn’t affected from the education of parents.^25^ In a similar study conducted by Deb at all. (2016) with 370 adolescents, it has been stated that domestic violence isn’t affected from the education of parents.^18^ While this finding complies with the result of the study performed by Kaplan et al (2014), Nabors et al (2006) and Kodan (2013), it isn’t in parallel with the findings of Efe and Ayaz (2010), Arat and Altınay (2008).^15,20,26-28^ Following the research, no significant difference was determined in terms of the violence tendencies of university students by the monthly income of the family. According to the result of the research conducted on university students by Kodan (2013), no significant difference was ascertained between the violence tendency scores of the participants and their monthly income level.^15^ They comply with the finding of the study performed by Merrill et al (2017).^29^ It has been stated that domestic violence attitude scores of university students don’t differ significantly by the average monthly income of the family. As a result of the study conducted by Birnbaum et al. (2017) in Canada with 353 children, it has been stated that domestic violence isn’t affected from the monthly income of the family.^25^ In a similar study conducted by Deb at all. (2016) with 370 adolescents, it has been stated that domestic violence isn’t affected from the monthly income of the family.^18^ Following the study of Kaplan et al. (2014), no significant difference was found between the attitudes of domestic violence against women by the monthly income of the family.^20^ According to the study conducted by Nabors et al. (2006) on college students, no significant difference was ascertained between the domestic violence attitudes by the monthly income of the family.^26^ It supports the finding of the research in this manner. However, many studies mention the relation between the income level and violence.^11,28,30^ In the study of Güler et al. (2005), 58.8% of women indicated the economic inefficiency as the most important reason increasing the domestic violence. These differences in the research findings may result from the characteristics of the research group. ^30^

It has been determined that 34.9% of the students (n=111) are subject to domestic violence, 7.9% (n=25) is rarely subject to violence, 42.8% (n=136) of them witness domestic violence and 11.9% of them (n=38) rarely witness it. In the study of Merrill et al. (2017) performed in Uganda with 499 staff working in school, the participants were stated to apply physical violence against the students with a rate of 43.1% (215) within the last week.^29^ In the study of Okour and Hijazi (2009) performed with 47102 university students, it has been stated that 41.6% of the students are subject to domestic violence and 68.1% of them witness domestic violence.^4^ In the study of Ergönen et al. (2007) conducted with the students studying in the field of health, 36.7% of them have been stated to experience physical violence in the family.^31^ According to another study carried out in Dokuz Eylül University(2009), 29% of a total of 192 senior students studying law have uttered that they are subject to domestic violence.^32^ In the study of Bryant & Spencer (2003) performed with 345 university students, it has been stated that 39% of the students have experienced any type of violence in the family in the last year. According to research findings, violence tendencies of university students being exposed to or witnessing violence are higher.^33^ Being subject to or witnessing domestic violence can be an important factor for individual’s exhibiting violent act. This situation can be explained with social learning; if parents are inclined to apply violence, the possibility of the child to accept violence increases and starts to use this solution in other cases encountered.^4^ Moreover, many studies indicate that the individual subject to violence also resorts to violence. In the research of Kodan (2013) carried out with the university students, violence tendencies of the students subject to violence were found to be higher.^15^ In the study of Okour and Hijazi (2009) conducted with 47102 university students, it has been stated that 49.4% of the students having violence tendency learn violence from their family.^4^ It has been determined in the research of Ayan (2007) that violence tendency of the students subject to violence is higher. The reasons for the student being subject to violence by their parents also constitute the reasons for aggressive tendencies of students. It has also been ascertained that 35.3% (n=66) of the university students are subject to physical violence, 32.6% (n=61) to verbal violence, 25.1% (n=47) to emotional violence and 7% (n=13) to economic violence. The most experienced type of violence was determined as “physical violence”.^12^ Following the research conducted by Kodan (2013), the type of domestic violence exposed by university students most was found to be emotional violence. Verbal violence is in the second place.^15^ In the study of Okour and Hijazi (2009) performed with university students, it has been determined that they are exposed to verbal violence (90.8%), physical violence (74.6%), emotional violence (62.2%) and economic violence (34.6%).^4^ It has been emphasized in the study of Güler et al. (2005) conducted on women that 59.7% of women have been subject to physical violence, 47.4% to verbal violence and 21.4% to emotional violence; however, women haven’t mentioned economic and sexual violence.^30^ In the study of Bryant & Spencer (2003) conducted with 345 university students, it is stated that 30% of the students are subject to physical violence, 23% to emotional and 0.3% to sexual violence^33^ Physical and verbal violence stand out most in the researchers conducted and this can be related to the fact that these types of violence are well-known and experienced more in the society. 

**Limitations: **It is based on voluntary participation of the students. They should attend the school on the dates when research data are collected. Conducting the study in only one region is limited and research results cannot be generalized to the population.

## Conclusion and Suggestions 

In this study, the phenomenon of violence directed to youth was conducted on the senior university students being a more developed group socially. Nevertheless, exposure of young people to violence was found to be high. However, their violence tendencies and domestic violence attitude scores were found to be low. Although the number of male students in the study is less than female students, the opinions of students regarding violence differed by sex. Violence tendencies and domestic violence attitude scores of male students were higher than female students. This study functioned as a source for other studies in which advanced analyses would be performed about violence. 

Domestic trainings should be provided to prevent violence. These trainings can change individuals' perception of violence and are also important in gaining problem solving skills. Therefore, the educational role of nurses in determining and preventing violence cannot be undeniable. In this regard, it is inevitable for nurses being in charge of the protection and improvement of community health to take responsibility in prevention of violence. Nurses have a special position in determining the children and families bearing the risk of violence, providing the necessary assistance to the child applying or being exposed to violence and finding acceptable solutions to the situation.
